# Optimal heat stress metric for modelling heat-related mortality varies from country to country

**DOI:** 10.1002/joc.8160

**Published:** 2023-07-12

**Authors:** Y. T. Eunice Lo, Dann M. Mitchell, Jonathan R. Buzan, Jakob Zscheischler, Rochelle Schneider, Malcolm N. Mistry, Jan Kyselý, Éric Lavigne, Susana Pereira da Silva, Dominic Royé, Aleš Urban, Ben Armstrong, Multi-Country Multi-City (MCC) Collaborative Research Network, Antonio Gasparrini, Ana M. Vicedo-Cabrera

**Affiliations:** 1 School of Geographical Sciences, University of Bristol, Bristol, UK; 2 Cabot Institute for the Environment, University of Bristol, Bristol, UK; 3 Climate and Environmental Physics, Physics Institute, University of Bern, Bern, Switzerland; 4 Oeschger Center for Climate Change Research, University of Bern, Bern, Switzerland; 5 Department of Computational Hydrosystems, Helmholtz Centre for Environmental Research GmbH—UFZ, Leipzig, Germany; 6 Φ-Lab, European Space Agency (ESA-ESRIN), Frascati, Italy; 7 Department of Public Health, Environments and Society, London School of Hygiene and Tropical Medicine, London, UK; 8 Centre on Climate Change & Planetary Health, London School of Hygiene and Tropical Medicine, London, UK; 9 Forecast Department, European Centre for Medium-Range Weather Forecast (ECMWF), Reading, UK; 10 Department of Economics, Ca' Foscari University of Venice, Venice, Italy; 11 Institute of Atmospheric Physics, Czech Academy of Sciences, Prague, Czech Republic; 12 Faculty of Environmental Sciences, Czech University of Life Sciences, Prague, Czech Republic; 13 School of Epidemiology & Public Health, Faculty of Medicine, University of Ottawa, Ottawa, Canada; 14 Air Health Science Division, Heatlh Canada, Ottawa, Canada; 15 Department of Epidemiology, Instituto Nacional de Saúde Dr Ricardo Jorge, Lisbon, Portugal; 16 Climate Research Foundation (FIC), Madrid, Spain; 17 Spanish Consortium for Research on Epidemiology and Public Health (CIBERESP), Spain; 18 Centre for Statistical Methodology, London School of Hygiene and Tropical Medicine, London, UK; 19 Institute of Social and Preventive Medicine, University of Bern, Bern, Switzerland

**Keywords:** climate and health, dry heat, heat stress, heat-related mortality, humid heat

## Abstract

Combined heat and humidity is frequently described as the main driver of human heat-related mortality, more so than dry-bulb temperature alone. While based on physiological thinking, this assumption has not been robustly supported by epidemiological evidence. By performing the first systematic comparison of eight heat stress metrics (i.e., temperature combined with humidity and other climate variables) with warm-season mortality, in 604 locations over 39 countries, we find that the optimal metric for modelling mortality varies from country to country. Temperature metrics with no or little humidity modification associates best with mortality in ~40% of the studied countries. Apparent temperature (combined temperature, humidity and wind speed) dominates in another 40% of countries. There is no obvious climate grouping in these results. We recommend, where possible, that researchers use the optimal metric for each country. However, dry-bulb temperature performs similarly to humidity-based heat stress metrics in estimating heat-related mortality in present-day climate.

## 1 Introduction

Humans are sensitive to high temperature conditions, and any sudden changes in heat conditions are a serious health threat to society. Heatwaves kill on massive scales at short timeframes (Buzan & Huber, 2020). In 2021, western North America experienced an extreme heatwave that was compounded by drought conditions (Thompson et al., 2022), leading to at least a thousand excess deaths (Philip et al., 2022). In contrast, the deadly 1995 Chicago heatwave was anomalously humid (Kunkel et al., 1996). Measuring heatwaves and determining the sources of environmental dangers is a complicated task. There is at least a 100-year literature on quantifying heat stress—the overwhelming of heat balance within the human body (Simon, 1993)—and who is at risk from such events (Buzan et al., 2015). The noisiness that makes determining the environmental dangers challenging is due to a plethora of physiological pathways, ranging from the physically healthy with excessive exercise (exertional metabolism), to ailments such as cardiovascular disease, diabetes, age and drug use (Bouchama et al., 2022; Ebi et al., 2021). This is further complicated by confounding socio-economic conditions such as the population distribution, access to cooling infrastructure and medical care (Reid et al., 2009).

Projections of different heat stress metrics increasingly diverge in the future, with the interquartile range in heat stress increases across regions and eight metrics being 2.1 to 3.6 K at 2 K global mean temperature increase, and 6.8 to 11.6 K at 6 K global mean temperature increase (Schwingshackl et al., 2021). These projected changes are robust within the coupled model intercomparison project (CMIP) (Buzan & Huber, 2020). Hence, there is a growing interest in determining the metric(s) that can best explain heat stress-related mortality.

Epidemiological studies that have examined the heat-mortality relationship mostly use daily mean dry-bulb temperature (T_mean_)—ambient air temperature measured by a thermometer that is unaffected by air moisture—as the exposure metric (Vicedo-Cabrera et al., 2021). The advantage of using this simple temperature measure is its easy interpretation and replicability. The lack of near-surface observational records for other meteorological parameters (e.g., solar radiation and wind speed) required for assembling other exposure measures is also a motivation for using T_mean_ alone. In studies where the effect of humidity is also considered, for example through the use of apparent temperature as the exposure metric (Armstrong et al., 2011), a weaker or similar statistical association with mortality has been found compared to T_mean_ (Anderson & Bell, 2012; Armstrong et al., 2011; Armstrong et al., 2019). In another study where temperature-humidity metrics are found to associate better with mortality than T_mean_ in some locations and age groups, these metrics’ predictive ability of mortality is similar to that of T_mean_ (Barnett et al., 2010). For these reasons, studies tend to quantify the association between T_mean_ and human mortality and then apply it to past observations or future projections, for the purpose of attributing past heat-related deaths to human-induced climate change (Vicedo-Cabrera et al., 2021), monitoring heatwave mortality in near real time (Lo et al., 2022), or projecting future impacts (Guo et al., 2018; Lo et al., 2019; Weinberger et al., 2017).

From a physiological perspective, evaporation of sweat helps regulate the human body temperature in hot conditions (Ebi et al., 2021). Atmospheric moisture is, therefore, also a contributing factor to heat stress. A recent study concludes that although soil droughts intensify heatwaves, they attenuate human heat stress by lowering air humidity (Wouters et al., 2022). Other recent climate research has focused on the wet-bulb temperature (T_w_)—the temperature that an atmospheric air parcel cools to from evaporation, or tacitly, the minimum achievable skin temperature from sweat evaporation—as a threat to human heat stress (Buzan & Huber, 2020). Studies have demonstrated that due to climate change, future projected atmospheric conditions may surpass critical thresholds for humans to cool themselves without mechanical aide (Pal & Eltahir, 2016; Parkes et al., 2022). Conversely, humans are dying from exposure to heat in present-day climate (Vicedo-Cabrera et al., 2021), and recent experimental work establishes that deadly T_w_ thresholds are likely to be much lower than previously thought (Vecellio et al., 2022).

Between T_mean_ and T_w_ lie a wide range of heat stress metrics that have been developed based on various thermal models of human comfort and empirical algorithms (Buzan et al., 2015). Among them are the commonly used apparent temperature (AT), which is a ‘feels like’ measure of temperature, humidity and wind speed; and discomfort index (DI), which is a combination of T_mean_ and T_w_. The former is used by the Australian Bureau of Meteorology in weather observations, whereas the latter is adapted by the Israeli Defense Force for decision making regarding heat stress (Buzan et al., 2015). National weather services elsewhere adopt other heat stress metrics (Buzan et al., 2015). A recent study finds that the Universal Thermal Climate Index (UTCI) is a suitable and similar indicator of temperature-related mortality in Europe, compared to T_mean_ (Urban et al., 2021).

Aside from these metrics, swamp coolers, that is, devices that consist of passing water through a moist membrane or other wet surfaces to achieve some degree of cooling, are often used to represent the capacity of evaporative cooling (Buzan et al., 2015). A swamp cooler that has 0% efficiency is not capable of evaporative cooling and results in a temperature that is the same as T_mean_. Conversely, a swamp cooler that has 100% efficiency achieves maximum evaporative cooling and results in T_w_. In reality, a typical swamp cooler has an efficiency between 65% and 80% (Buzan et al., 2015).

Despite the availability of heat stress metrics in the literature, few studies have attempted to systematically determine a primary factor in modelling heat-related mortality for different locations (Mora et al., 2017). One study investigated the use of heat indices on heatwave mortality in a state in the USA (Kent et al., 2014). Another study highlighted the need to use sophisticated heat stress metrics in future climate-health projections but did not attempt to compare the modelling power of the metrics (Vanos et al., 2020). Calculating these metrics correctly at appropriate temporal frequencies without making approximations is tricky, as suggested by the limitations of previous research (Dunne et al., 2013; Russo et al., 2017; Schwingshackl et al., 2021). Combining this complexity with available mortality data, temporal frequency and local versus regional locality tags leave a muddied picture for epidemiologists to work with. However, from the climate perspective, there have been great improvements in the availability and resolution of climate data. Sophisticated techniques have led to heat stress diagnostic batteries (Buzan et al., 2015; Casanueva et al., 2019) to analyse heat conditions. Furthermore, focus on higher temporal frequencies and detailed calculation methods improve accuracy of assessments and projections (Buzan & Huber, 2020).

In this study, we systematically assess the associations between temperature and heat stress metrics with human mortality. Using climate data from the ERA5 reanalysis (Hersbach et al., 2020) and daily all-cause mortality data from the Multi-Country Multi-City (MCC) Collaborative Research Network (London School of Hygiene and Tropical Medicine, 2021), we establish warm-season exposureresponse associations for 604 locations in 39 MCC-defined countries with two-stage time series analyses and Distributed Lag Non-linear Models (DLNMs). This represents the state-of-the-art method in climate epidemiology (Armstrong et al., 2011; Gasparrini & Armstrong, 2013; Gasparrinia et al., 2010; Guo et al., 2014; Ma et al., 2014; Petkova et al., 2014). We statistically compare the model fit between eight exposure metrics: T_mean_, a commonly used metric in epidemiological studies; T_w_, a metric increasingly used in climate projections; AT and DI, metrics adopted by national governments and swamp coolers at 20%, 40%, 60% and 80% efficiencies (denoted here as Swmp20, Swmp40, Swmp60 and Swmp80), that is, moist thermodynamic metrics that are fundamentally tied to atmospheric buoyancy. We examine metrics tied to atmospheric buoyancy because during local warm seasons, the atmosphere over most land masses is close to moist adiabatic most of the time (Buzan & Huber, 2020). Comparing these metrics allow us to tie different commonly used heat stress metrics to atmospheric conditions robustly, and we attempt to find the metric of best fit for each country for the purpose of informing national heat-health action plans. We investigate the differences using the best-fit metrics makes in terms of warm-season heat-related mortality estimation. To our knowledge, our study is the first to examine the suitability of heat stress metrics in a global multi-city, multi-country setting. Our results have important implications for attributing past mortality to heat stress, as well as projecting future mortality in climate change scenarios.

## 2 Methods

### 2.1 Mortality data

We use daily all-cause mortality data from the MCC Collaborative Research Network (London School of Hygiene and Tropical Medicine, 2021) throughout this analysis. The MCC database is the most comprehensive epidemiological dataset of its kind (Mitchell, 2021; Vicedo-Cabrera et al., 2021), and it is continuously updated and extended. The version of MCC data (version 2020-09-07) used in this study covers 734 locations in 43 MCC-defined countries, spanning the period 1969–2018. Detailed description of the MCC data, including all location names, and the total mortality count in each location and missing data, can be found in the supplementary document of a recent publication (Vicedo-Cabrera et al., 2021).

We select a subset of these locations, that is, a total of 604 locations in 39 countries, where mortality data are available at the city or district level, rather than the coarser regional or province level. This choice is motivated by the need to align temperature and heat stress metrics, derived from high-resolution climate reanalysis data (see below), with mortality observations. We also subset the mortality data in the time dimension by selecting data from 1979 onwards, to align them with the climate reanalysis data period, which started in 1979 at the time of research. Table S1 in Supplementary Information shows the number of locations and the time period used for each MCC-defined country in this study.

### 2.2 Temperature and heat stress metrics

All metrics, that is, dry-bulb temperature (T_mean_) and the seven selected heat stress metrics, are derived from daily mean meteorological variables available from the ERA5 reanalysis (Hersbach et al., 2020). Daily mean values are used to ensure that all the meteorological variables are temporally consistent, and that they are at the same time (daily) resolution as the mortality data. Ideally, sub-daily instantaneous and contemporaneous data should be used to compute heat stress due to its non-linear nature (Buzan & Huber, 2020). We discuss the implications of using daily means in Section 4.

T_mean_ at 2 m height, daily mean dew point temperature at 2 m height (T_dew_), daily mean surface air pressure and daily mean eastward and northward components of wind at 10 m height were obtained from ERA5 for the selected 604 MCC locations. We derive vapour pressure (e_RH_) from T_dew_, and relative humidity (RH) from T_mean_ and T_dew_ (Li, 2019). Note that the RH calculations do not represent actual daily mean RH, but instead provide reasonable approximations using available data. We calculate 10 m wind speed (u_10m_) from the eastward and northward components of wind. The calculation of the seven heat stress metrics is detailed below.

We derive T_w_ (in °C) from daily mean T_mean_, RH and surface air pressure following the Davies–Jones method (Davies-Jones, 2008). This is achieved by using an opensource python script, WetBulb.py (Li, 2019), which is based on a set of peer-reviewed routines for heat stress calculation called HumanIndexMod (Buzan et al., 2015).

We calculate apparent temperature (AT; in °C) from T_mean_ (in °C), vapour pressure (e_RH_; in Pa) and wind speed at 10 m height (u_10m_; in m/s) following the relevant equation in the literature (Buzan et al., 2015). Note that these AT calculations do not provide actual daily mean AT, but are reasonable approximations.

$$AT=T_{mean}+\frac{3.3e_{RH}}{1000}-0.7u_{10m}-4$$

Discomfort index (DI; in °C) is a heat stress metric adopted by the Israeli Defense Force and the Israeli Weather Bureau for decision making. Its original conception was for calculating degree cooling days for air conditioning (Buzan et al., 2015). It is a combination of T_mean_ and T_w_, with the following equation (Buzan et al., 2015):

$$DI=0.5T_{mean}+0.5T_{w}$$

Finally, we calculate evaporative cooling efficiency temperatures, colloquially known as ‘swamp coolers’, at 20%, 40%, 60% and 80% efficiencies (Swmp20, Swmp40, Swmp60 and Swmp80; in °C) according to the following equation (Buzan et al., 2015):

$$Swmp_{\eta }=T_{mean}-\frac{\eta }{100}\left(T_{mean}-T_{w}\right)$$

Where η is 20, 40, 60 or 80, depending on the cooler efficiency. Swamp coolers cool a person down to a target temperature, denoted here as Swmpη, through evaporative cooling (Buzan et al., 2015). We use the swamp cooler temperatures studied here, Swmp20 to Swmp80, to systematically evaluate evaporative resistance (Grimmond & Oke, 1991). For instance, at 20% efficiency evaporative resistance is high and there is little humidity modification in the target temperature (Swmp20) from T_mean_. The opposite is true at 80% efficiency. The swamp cooler temperatures, therefore, allow us to characterize the aforementioned battery of heat stress metrics to different types of moist thermodynamic conditions of the atmosphere and contextualize their relationships to mortality outcomes.

### 2.3 Two-stage time series analysis

For each selected MCC location, we apply a two-stage time-series approach to derive location-specific exposure-mortality associations. In the first stage, we perform location-specific time series analysis with DLNMs and quasi-Poisson regression to derive the exposure-response association between each included exposure metric and all-cause mortality over the four warmest consecutive months (warm season) in each location (Vicedo-Cabrera et al., 2021). DLNMs account for delayed and non-linear effects of time-varying exposures and quantify the net effects over a pre-defined lag period. Following the DLNM methodology, the exposure-response function is modelled with a quadratic B-spline function, with internal knots at the 50th and 90th percentiles of location-specific, observed warm-season temperature or heat stress range. A lag period of 10 days is considered and modelled with a natural spline with two internal knots at equally spaced values in the log scale. The model controls for seasonality and long-term trends by including an indicator for day of week, a natural spline function of 4 degrees of freedom for day of the year, an interaction term of this spline with year, and another natural spline with 1 degrees of freedom per decade. This is a standard set up in the literature (Gasparrini, Guo, Hashizume, Kinney, et al., 2015). The resulting bi-dimensional set of coefficients from each location are then reduced across the lag dimension into an overall cumulative exposure-response curve representing the association between heat stress and mortality across the 10-day lag period.

In the second stage of the analysis, we pool the derived location-specific exposure-response curves in a multivariate meta-regression model (Gasparrini et al., 2012). This approach provides improved estimates of the heat-mortality associations at the location level, defined as best linear unbiased predictions (BLUPs). BLUPs borrow information across units within the same hierarchical level and offer more accurate estimates, especially in locations with limited statistical power. We include as meta-predictors the country-level gross domestic product, location-specific average temperature or heat stress and its interquartile range, and indicators of climatic classification in the Köppen–Geiger classification system (Kottek et al., 2006). This follows the same meta-regression model used in a previous study (Vicedo-Cabrera et al., 2021). The location-specific associations defined by the BLUPs are used in the quantification of the heat-related mortality impacts, following the method previously described (Gasparrini & Leone, 2014). By using flexible but smooth curves for exposure-response associations and BLUPs to borrow strength across locations, our methods allow for more precise and unbiased mortality estimates in extremes conditions, which statistically occur less frequently than milder conditions, than other approaches (Gasparrini, Guo, Hashizume, Lavigne, et al., 2015).

For each location and exposure metric, we compute the daily number of heat-related deaths based on the corresponding daily temperature or heat stress value, total mortality, and the estimated exposure-mortality association. We sum daily heat-related deaths across all days in the warm season, when temperature or heat stress is above the minimum mortality temperature (MMT), which corresponds to the optimal temperature or heat stress for which the mortality risk is minimum. Separately for each exposure metric, we then sum the estimated warm-season heat-related deaths across locations within the same countries. Impacts are expressed as fractions of deaths attributable to heat stress (in %), corresponding to the percentage of heat-related deaths over total mortality. Empirical 95% confidence intervals are estimated from Monte Carlo simulations of the coefficients defining the BLUPs, assuming a multivariate normal distribution. These correspond to the 2.5th and 97.5th percentiles of the empirical distribution.

### 2.4 Goodness-of-fit comparison

We use the Quasi–Akaike Information Criterion (qAIC) (Gasparrinia et al., 2010) to assess the goodness-of-fit of each location-specific exposure-response model between the eight exposure metrics and mortality. qAIC assesses the quality of a fitted model relative to other models through the quasi-log-likelihood function and is frequently used in epidemiological studies to assess the goodness-of-fit in time-series analyses (Armstrong et al., 2019; Gasparrinia et al., 2010). For each exposure metric, we calculate qAIC from the first stage of the time series models (Section 2.3), and then sum the qAIC values across all locations within the same MCC-defined countries to obtain country-level results, following a previously adopted approach (Armstrong et al., 2019). We then identify the metric that gives the lowest qAIC value in each country as the best-fit metric to warm-season heat-related mortality. At the country level, we compute the difference in qAIC value (ΔqAIC) between each studied exposure metric and the best-fit metric, in order to compare the metrics’ goodness-of-fit. The same comparative approach has been used in a recent study (Mistry et al., 2022).

## 3 Results

### 3.1 Exposure metrics in the temperature-humidity space

**Figure 1:**
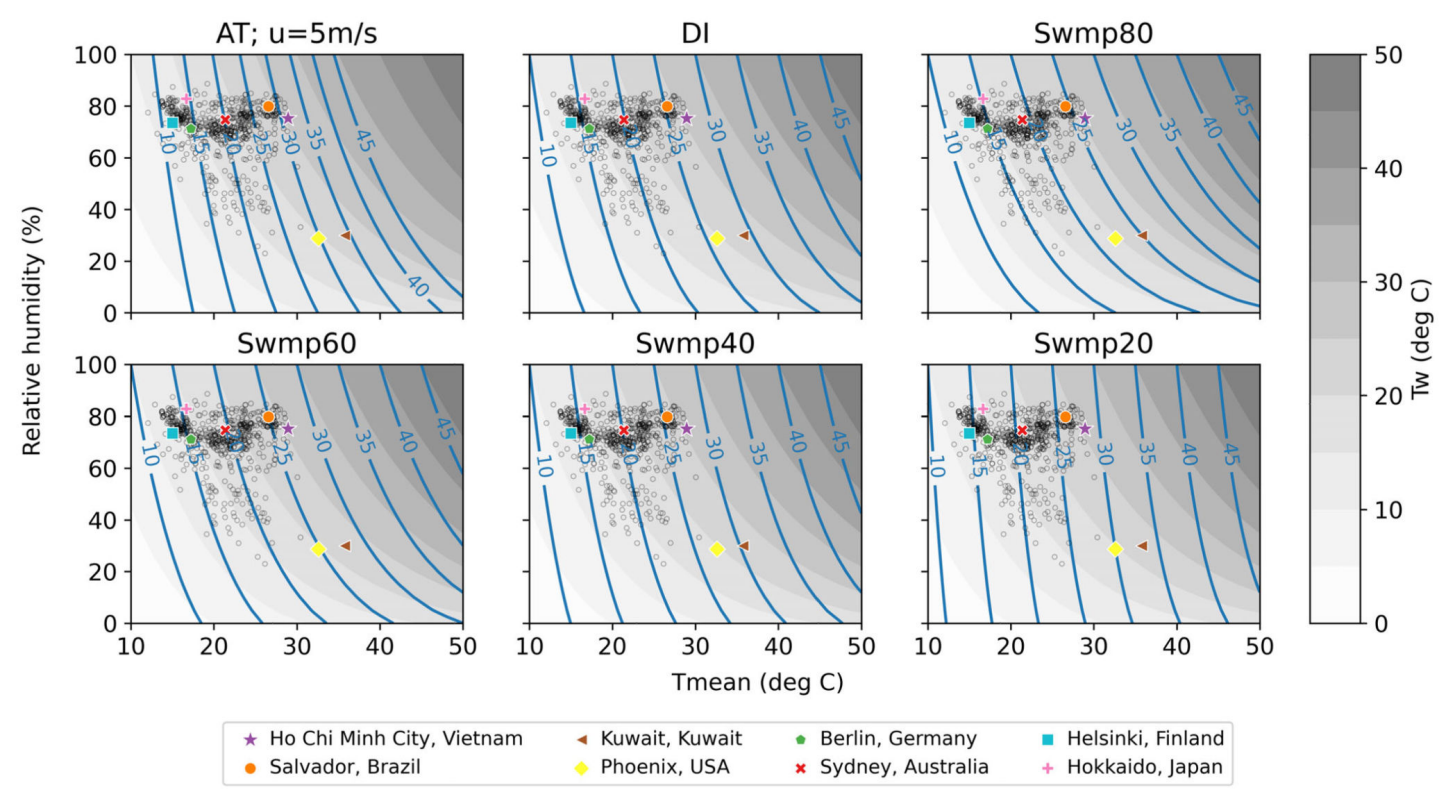
Temperature-humidity state diagrams of the heat stress metrics. The blue lines and grey shading indicate isopleths of the metrics. AT is apparent temperature when 10 m wind speed is 5 m/s, DI is discomfort index, whereas Swmpη is swamp cooler temperature at η% efficiency. Grey shading shows T_w_, that is, wet-bulb temperature, in °C, assuming standard atmospheric pressure. The grey open circle markers indicate the average warm-season climates in 604 MCC locations. The coloured markers indicate the average warm-season climates in eight locations that are representative of Köppen–Geiger climate zones—Equatorial: Ho Chi Minh City and Salvador; Arid: Kuwait and Phoenix; Warm temperate: Berlin and Sydney and Snow: Helsinki and Hokkaido.

We start by examining how the exposure metrics vary with dry-bulb temperature (T_mean_) and relative humidity. The blue lines in Figure 1 indicate equal values of the studied metrics at 0–50°C T_mean_ (x-axis) and all possible relative humidity (y-axis). Since T_mean_ is independent of air moisture, lines connecting equal T_mean_ values would appear as vertical lines if drawn. T_w_ (grey contours in Figure 1) and the swamp cooler temperatures all have the property of having the same value as T_mean_ at 100% relatively humidity, as evaporative cooling cannot take place when ambient air is saturated with water vapour. Since DI is defined as the average of T_mean_ and T_w_, it is also equal to T_mean_ at 100% relative humidity. This is not true for AT, however, as it is also dependent on wind speed and represents the deviation from a reference comfort zone (top left panel in Figure 1). Overall, the lower the ambient relative humidity, the more the value of the heat stress metrics deviates from the value of T_mean_ but to different extents.

T_w_ deviates from T_mean_ the most, showing the strongest dependence on humidity. This means recent climate studies incorporating T_w_ have mainly focused on a heat stress metric with a large humidity modification. We examine how well high T_w_ translates into warm-season mortality outcomes later in this study. Between T_mean_ and T_w_ lie the rest of the metrics, with Swmp20—the target temperature of a swamp cooler with only 20% efficiency—being most similar to T_mean_, and Swmp80 being most similar to T_w_. We refer to metrics that have no or little humidity modification (i.e., T_mean_ and Swmp20) as ‘dry heat’ metrics hereafter, whereas metrics with a large humidity modification (i.e., T_w_ and Swmp80) are referred to as ‘humid heat’ metrics. The other commonly used metrics such as AT and DI lie somewhere near the middle of this dry heat-humid heat spectrum.

The average warm-season climates (over the corresponding data periods, see Table S1 in Supplementary Information) of all included MCC locations (grey open circles in Figure 1) and eight selected locations representing different Köppen–Geiger climate zones (Kottek et al., 2006) and continents (coloured markers) are also shown in Figure 1. According to the Köppen–Geiger classification, Ho Chi Minh City in Vietnam and Salvador in Brazil have equatorial climates, whereas Kuwait in Kuwait and Phoenix in the USA represent arid climates. Berlin in Germany and Sydney in Australia both have warm temperate climates, whereas Helsinki in Finland and Hokkaido in Japan are in the snow climate zone.

Figure 1 demonstrates that warm-season mean climates vary both within and across climate zones, but to different extents. Unsurprisingly, the arid climates of Kuwait and Phoenix stand out as the hottest and driest among the eight representative locations. A vast majority of the rest of the locations have substantially different warm-season average T_mean_ that are representative of their climates but similar relative humidity that lie between 70% and 85% (Figure 1). This shows that variability in T_mean_ in the warm season is much larger than the corresponding variability in humidity across the MCC locations, even though they are located in different climate zones.

### 3.2 Exposure-response associations

**Figure 2:**
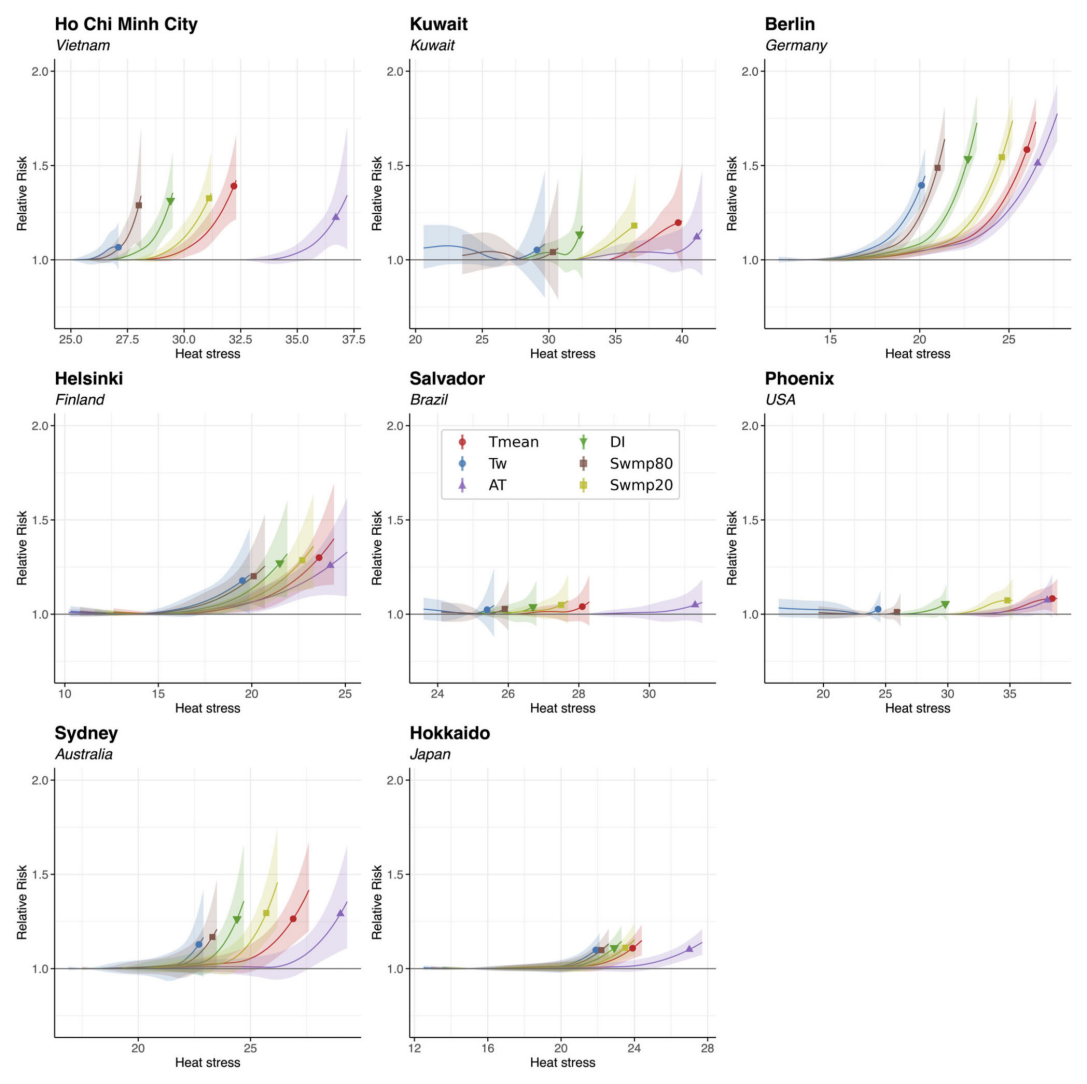
Exposure-response associations at representative MCC locations. The x-axes show the absolute values of T_mean_ (red), T_w_ (blue), AT (purple), DI (green), Swmp80 (brown) and Swmp20 (olive) at the locations, and the y-axes show the corresponding relative mortality risk (RR). The markers (circles for T_mean_ and T_w_, upward triangle for AT, downward triangle for DI and squares for Swmp80 and Swmp20) indicate the relative risk at the 99th percentile of the corresponding heat stress metric distribution in the warm season. Shading represents the 95% empirical confidence interval.

For the same representative locations, Figure 2 shows the exposure-response associations between six of the studied exposure metrics and all-cause mortality in the warm season. The associations for Swmp40 and Swmp60 are not shown because they are similar and lie between that of Swmp20 and Swmp80. We show Swmp20 and Swmp80 in Figure 2 to represent the extreme ends of evaporative cooling efficiency. In general, the risk of mortality increases with exposure to heat or heat stress above certain thresholds, as reported in the literature (Vicedo-Cabrera et al., 2021).

Exposure metrics that are similar in the temperaturehumidity space (as shown in Figure 1) have similar relative mortality risk curves. This is particularly evident by the T_mean_ (red) and Swmp20 (olive) curves, and the T_w_ (blue) and Swmp80 (brown) curves. The markers in Figure 2 indicate the relative risk of mortality at the 99th percentile of the respective warm-season temperature or heat stress distribution at each representative location. Thus, they represent mortality risks at extreme warmseason exposure to heat stress. In Ho Chi Minh City, Kuwait, Phoenix, Berlin, Sydney, Helsinki and Hokkaido, either T_mean_ or Swmp20 gives the highest relative mortality risk at the 99th percentile among the studied metrics. This suggests that extreme exposure to dry heat, compared to humid heat, poses a higher risk to human life in these places. In Salvador, AT gives the highest mortality risk at the 99th percentile.

### 3.3 Best-fit exposure metrics for warmseason mortality

Nevertheless, an exposure metric that is associated with the highest mortality risk is not necessarily a metric that statistically fits the underlying mortality observations best. We quantify the goodness-of-fit of each exposure-response association using the qAIC (Burnham & Anderson, 1998). For each of the 39 MCC-defined countries that are included in this study, we sum the locationlevel qAICs within the same country and compare the resulting qAIC value between the eight exposure metrics. The smaller the qAIC value, the better the model fit. We identify the metric that gives the smallest qAIC value as the ‘best-fit’ metric for predicting warm-season mortality.

**Figure 3:**
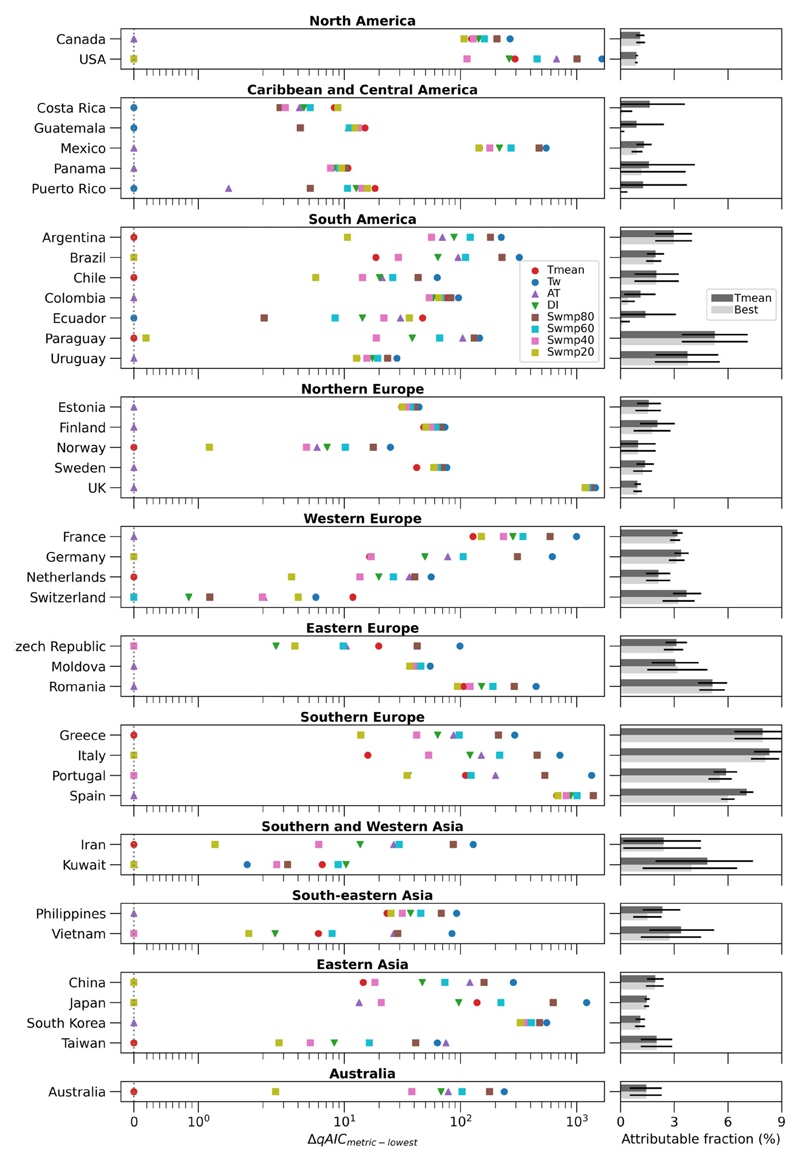
Country-level best-fit metrics, their performance in model fit compared to the other metrics, and the implications on mortality. Left panel: ΔqAIC between each studied metric and the metric that has the lowest qAIC value for all studied MCC-defined countries, grouped by United Nations regions. The larger the ΔqAIC is for a metric, the worse it is in terms of modelling warm-season heat-related mortality compared to the best-fit metric for the same country. Right panel: fractions of warm season deaths attributable to heat, estimated with T_mean_ (dark grey bar) and each country’s best-fit metric (light grey bar). Error bars show 95% confidence intervals.

In the left panel of Figure 3, we show the best-fit metric for each of the 39 included MCC-defined countries, grouped by regions defined by the United Nations (United Nations Statistics Division, 1999). The x-axis of this panel shows the difference in qAIC value between each exposure metric and the best-fit metric, denoted as ΔqAIC. By this definition, the metric at ΔqAIC = 0 (dotted line in figure panel) for each country is its best-fit metric. Our results show great heterogeneity in best-fit metric among the included countries. Dry heat metrics, that is, T_mean_ and Swmp20, are best-fit for modelling warm-season heat-related mortality in 23% and 18% of the studied countries, respectively. Specifically, they are dominant in studied locations in Southern and Western Asia, Eastern Asia and Australia.

On the other hand, humid heat (T_w_) is best-fit for warm-season mortality in Caribbean, Central or South American countries—Costa Rica, Guatemala, Puerto Rico and Ecuador—accounting for 10% of the studied countries. Although also considered a humid heat metric that is similar to T_w_ (Figure 1), Swmp80 is not found to be a best-fit metric for any studied country.

Towards the middle of the dry heat-humid heat spectrum (Figure 1), AT, a metric combining temperature, humidity and wind speed, is the best-fit exposure metric for nearly 40% of the studied countries. Figure 3 shows that it is the dominant metric in Northern Europe and Eastern Europe, which are by far the best-mapped regions in the MCC dataset (Table S1) and include a lot of small countries. This explains the high proportion of countries having AT as their best-fit metrics and may not be reflective of the global picture.

Despite being a commonly used heat stress metric, DI, which is the average between T_mean_ and T_w_, is not a best-fit metric in any of the countries. Conversely, Swmp40 is best-fit for modelling warm-season heat-related mortality in Czech Republic, Portugal and Vietnam (8% of the studied countries); whereas Swmp60 is best-fit for Switzerland (~3% of the countries).

Up until now, it is unclear how the model fits of the other exposure metrics are compared to the best-fit metric in each country. We use the ΔqAIC of the rest of the metrics to indicate this (left panel of Figure 3). For any exposure metric, the larger its ΔqAIC is relative to the best-fit metric, the worse its performance is compared to that of the best-fit metric, and vice versa. We find large ΔqAICs in a number of countries, indicating where the rest of the metrics perform noticeably worse than the corresponding best-fit metric. In other words, the best-fit metric performs noticeably better in terms of modelling warm-season heat-related mortality in these countries.

We find that T_mean_ performs better than the rest of the metrics for Argentina and Greece; whereas Swmp20 performs better than the rest of the metrics for the USA, Brazil, Germany, Italy, China and Japan. AT performs better than the rest of the metrics in Canada, Mexico, Colombia, Uruguay, Estonia, Finland, Sweden, the UK, France, Moldova, Romania, Spain, Philippines and South Korea. Finally, Swmp40 performs better than the rest of the metrics for Portugal. For the countries not mentioned here, their respective best-fit metrics perform only marginally better than the other metrics (note the log scale on the x-axis of the left panel of Figure 3). This means that even though humid heat (T_w_) is found to be best-fit for warm-season mortality in Costa Rica, Guatemala, Puerto Rico and Ecuador, the other metrics give similar model fits to the mortality data.

Given that most epidemiological studies to date use T_mean_ as the exposure metric to estimate heat-related mortality, we compare country-level warm-season heat-related mortality estimated with the best-fit metric versus that with T_mean_ in the right panel of Figure 3. The x-axis of this panel shows the fraction of all warm-season allcause deaths in a country that is attributable to heat stress. The error bars show the 95% confidence intervals of attributable fraction. These attributable fractions and confidence intervals are also listed in Table S2.

In general, using the respective best-fit metric results in a similar attributable fraction, compared to using T_mean_. This holds true for countries where the best-fit metric performs noticeably better than T_mean_, as indicated by a large ΔqAIC_T_mean_-lowest_. For example, Swmp20 is found to be the best-fit metric for the USA and it estimates an attributable fraction of 0.88% (95% confidence interval: 0.80–0.96%). For comparison, T_mean_ estimates an attributable fraction of 0.9% (confidence interval: 0.82–0.97%). AT is the best-fit metric for the UK and it estimates an attributable fraction of 0.96% (confidence interval: 0.71–1.20%). This is also comparable to the 0.94% (confidence interval: 0.77–1.13%) estimated with T_mean_. The only exception we find is for Spain, whose best-fit metric (AT) estimates a lower attributable fraction (6%; confidence interval: 5.61%–6.38%) than the 95% confidence interval of that from T_mean_ (7.05%; confidence interval: 6.68%–7.42%).

These results are further demonstrated in Figure S1 in Supplementary Information, where the difference in attributable fraction between the best-fit metric and T_mean_ is shown for selected countries that have a large ΔqAIC_T_mean_-lowest._ We note that our attributable fractions, represented by the area under the exposure-response curves between the heat stress value of minimum mortality risk (MMT) and extreme heat stress (Figure 2), are dependent on the percentile at which the MMT is located within its climatological warm-season distribution. This percentile may differ between the exposure metrics for the same locations, leading to less constrained attributable fraction differences in some cases.

## 4 Discussion

This study addresses two questions: (i) ‘which heat stress metric best models warm-season heat-related mortality outcomes in different countries in recent decades?’, and (ii) ‘how does it compare with other metrics in terms of model fit and heat-related mortality impacts?’. The heterogeneity found in our results highlights the importance of considering heat stress exposure metrics on a country-by-country basis. We emphasize that our results are reflective of the cities and countries sampled in the MCC dataset, which, despite being the most comprehensive dataset of its kind in the world, represents European, North American and some East Asian countries (e.g., Japan and South Korea) far better than other regions (Table S1).

We find T_mean_ to be best-fit and far better than AT for modelling warm-season heat-related mortality in Australia (Figure 3), even though AT was specifically developed for climates in Australia and is still used to indicate the ‘feels like’ temperature by the Australian Bureau of Meteorology (Buzan et al., 2015). This suggests that a heat stress metric developed for a certain place is not necessarily best for modelling heat-related mortality in that place, potentially because of physiological factors that are not accounted for in the metric. For instance, AT was designed around a thermo-physiology model based on a healthy, ‘typical human’ who is 1.7 m tall and weighs 67 kg (Anderson et al., 2013; Steadman, 1979), which may not be representative of the more vulnerable population.

For countries including the UK but excluding the USA, we find AT to be the best-fit metric (Figure 3). This is partially inconsistent with the literature, which has reported a similar effect or weaker fit for AT compared to T_mean_ for England and Wales (Armstrong et al., 2011), the USA (Anderson & Bell, 2012), and a multi-country average (Armstrong et al., 2019). We suggest two possible reasons for the inconsistency with the aforementioned UK study. First, this previous UK study was based on regional data from the period 1993–2006 (Armstrong et al., 2011), whereas our study includes data for smaller conurbations up until 2016 (Table S1), hence representing updated exposure-response associations. Second, the previous study used a simplified version of AT that is based only on temperature and humidity (Anderson et al., 2013; Basu & Ostro, 2008), whereas we use a version of AT that also considers the effect of wind. For the USA, we find Swmp20, a ‘dry heat’ metric that has little humidity modification, to be the best metric. This is consistent with the previous USA study, which used similar data to ours (both ending in the 2000s). It is unclear from the literature whether this USA study incorporated wind speed in their AT calculation (Anderson & Bell, 2012; Kalkstein & Valimont, 1986). Since our study focuses on the country level, we cannot compare our results with the multi-country average study. A lack of previous studies that have considered the other heat stress metrics, limits direct comparison of our other results with the literature.

Using England as a case study, recent research proposed that a DLNM-based framework, coupled with near-real time T_mean_ observations, can be used for heat-wave mortality monitoring as part of national heat-health action plans (Lo et al., 2022). Our results for the UK show that AT associates with warm-season mortality outcomes better than T_mean_ (Figure 3), but that it estimates a similar fraction of attributable deaths (see Figure 3; Figure S1). Therefore, although AT is a better metric for heatwave mortality monitoring in England, current available data suggest that replacing T_mean_ with AT in the monitoring framework will not substantially affect the estimation of the current impact of extreme heat on public health. This is, however, not the case for Spain, for which AT is the best-fit metric and substantially fewer heat-related deaths are found with AT than T_mean_.

Extreme heatwaves are often preceded by or coincide with anomalous drought conditions, as dry soils exacerbate air temperatures through increased sensible heat and reduced latent heat (Wehrli et al., 2019). Since ‘dry heat’ metrics with no or little humidity modification (T_mean_ and Swmp20) are found to dominate over ‘humid heat’ metrics with large humidity modification (T_w_ and Swmp80) as proxies of warm-season mortality (Figure 3), our results suggest that hot-dry compound events are more dangerous to human life than found in a recent study based on a heat stress metric not studied here (Wouters et al., 2022).

While T_w_ is undeniably associated with human mortality (Figure 2), it is found to be best-fit for warm-season heat-related mortality only in Costa Rica, Guatemala, Puerto Rico and Ecuador (Figure 3), all of which have relatively short mortality records (Table S1) and therefore unstable exposure–response associations. This is reflected by the large confidence intervals in attributable fraction across all studied metrics in these countries (Figure S2 in Supplementary Information). Furthermore, in none of these countries is T_w_ noticeably better than the rest of the metrics at modelling mortality in recent history. This may be because (i) T_w_ is a complex metric by definition, leading to uncertainty in its observations; (ii) deadly T_w_ thresholds have barely been reached in present-day climate (Im et al., 2017; Raymond et al., 2020; Raymond et al., 2021; Sherwood & Huber, 2010) and (iii) high humidity negatively affects human thermal perception (Buonocore et al., 2018; Quinn & Shaman, 2017), leading to earlier intervention than in drier conditions. Researchers should take note of our result about T_w_ in heat-mortality research. We note, however, that these relationships may change in a changing climate.

The relationships between heat stress and mortality also vary within countries, particularly in larger countries such as China and the USA. By aggregating to the country level our results have implications for national heat-health action plans (Lo et al., 2022), but we have not been able to demonstrate a higher level of granularity, which is important to reveal vulnerability and inequality within the populations. Aggregation of goodness of exposure–response model fit across different locations within the same countries has been done through summing qAIC values. While this approach is based on published work, alternative ways of assessing aggregated model fit could be explored through simulations in future work. We encourage researchers to explore the theoretical basis for defining combined goodness of fit and examine higher granularity within individual countries in future work.

This work has focused on mortality, but heat exposure is not only associated with mortality but also other health risks. Previous studies have found humid heat stress to be a major concern for occupational health in sugarcane harvesters in Costa Rica (Crowe et al., 2013), a country for which we find T_w_ to be the best-fit metric for warm-season heat-related mortality, albeit only being marginally better than the other metrics (Figure 3). Humid heat stress is also associated with chronic pain (Dixon et al., 2019) and is a better indicator for kidney stone presentation in patients than T_mean_ (Ross et al., 2018). Humidity is, therefore, an important driver of heat-health risks, even though its contribution to heat stress does not currently lead to better modelling of mortality outcomes. We recommend that researchers conduct analyses like this one on other health outcomes and chronic exposures.

This study has compared eight exposure metrics that are either commonly used in the heat stress literature (T_mean_, T_w_, AT and DI) or representative of a wide range of evaporative cooling capacities (Swmp20 to Swmp80). While we believe that the selected metrics cover a wide range in the temperature-humidity space (Figure 1), other heat stress metrics such as the UTCI (Urban et al., 2021), Heat Index and Humidex (Buzan et al., 2015) exist. We note that AT, UTCI and Heat Index are all derived from mechanistic thermo-physiology models that use a typical person of a certain weight, wearing shorts and a t-shirt and being sedentary in the shade (Buzan et al., 2015) or walking outdoors at 4 km/h (Fiala et al., 2012; Jendritzky et al., 2012) as boundary conditions. Therefore, UTCI and Heat Index represent a limited population similar to that represented by AT, one of our included metrics. Because of their similar origins, Heat Index values are consistent with AT at a range of weather conditions, with exceptions at <10% relative humidity (outside the range of climates in this study, Figure 1) (Anderson et al., 2013). Nevertheless, future work could include these additional metrics for an even more comprehensive comparison. As mentioned above, subtlety also exists in the calculation of metrics of the same name (e.g., with or without the consideration of wind speed in AT).

Furthermore, we have based our study on daily heat stress values because they are temporally consistent for comparison and that they have the same temporal resolution as the mortality data. Doing so is a major improvement from previous studies that used coarser temporal resolutions (e.g., monthly means) or compared metrics derived from incomparable timings (e.g., daily maximum temperature versus heat stress derived from daily average temperature and humidity). However, we acknowledge that daily temperature or heat stress extremes may be more relevant for health outcomes in places with high climate variability. Calculating daily extremes would require higher-resolution instantaneous data for all studied locations (Buzan & Huber, 2020). Future work should extend our work with such data and examine their associations with human mortality outcomes, as the results can have important policy implications.

Although gridded climate data such as the ERA5 reanalysis (Hersbach et al., 2020) have been shown to estimate similar temperature-mortality associations and impacts compared to weather station data in the 39 countries studied here (de Schrijver et al., 2021; Mistry et al., 2022), their resolutions are not high enough to resolve urban and rural differences in air temperature, moisture, wind and solar radiation, all of which affect human heat stress (Buzan et al., 2015). For instance, low wind conditions in an urban area tend to lead to higher T_mean_ than its surroundings (the urban heat island effect, or UHI), both of which are contributing factors to high AT. Resolving these metrics at a higher spatial resolution than ERA5 may lead to different results regarding their goodness of fit with mortality. UHIs have been shown to be responsible for ~4% of summer mortality in Europe (Iungman et al., 2023), so not capturing them in our climate data may have led to an underestimation of the mortality impact in major cities. We acknowledge that reanalysis data other than ERA5 and weather station data can be used in future work, and that results may be different from these data. The European Commission has recently launched the Destination Earth (DestinE) initiative to build multiple digital twins of the Earth at a higher spatio-temporal resolution (i.e., 1 km horizontal resolution and hourly time resolution). The first two digital replicas are on weather-induced hazards (e.g., heatwaves) and climate change adaptation, both focused on addressing weather changes at the local level (European Commission, 2022). Urban and rural differences will be better resolved in this new dataset.

Finally, some countries included in this study have short daily mortality records (e.g., 5 years in Ecuador, the Philippines, Uruguay and Vietnam; see Table S1), leading to large uncertainties in our results. Africa is not included at all due to a lack of mortality data. This limitation prevents this study from being a global study and is a major obstacle to heat-mortality research (Mitchell, 2021). Future work should repeat this study on the global scale when more data become available.

In addition to looking at the past, it is important to apply present-day exposure–response associations to future climate change projections for preventative adaptation and mitigation measures. Many studies have done so using T_mean_ already (Guo et al., 2018; Lo et al., 2019; Weinberger et al., 2017). Given that the heat stress metrics studied here vary in their associations with present-day mortality, and that the metrics themselves are expected to diverge in the future as a function of global mean warming (Schwingshackl et al., 2021), future work should investigate the implications of this divergence for projected mortality levels. For example, previous work demonstrated similar future temperature and AT trends (Schwingshackl et al., 2021), implying that these metrics may give similar projected mortality in Northern and Eastern Europe, where AT is predominantly the best-fit metric. However, the same study found lower future T_w_ than temperature trends in all regions, including in Central America. This implies that future T_w_-related mortality in Costa Rica, Guatemala, Ecuador, and potentially Puerto Rico (where T_w_ is found to be best-fit) may differ from future T_mean_-related mortality. Furthermore, whether preventative adaptation actions should be based on mortality levels that are projected with the metric of best-fit (according to present-day situations) or the metric of highest impact, warrants an in-depth discussion in future work.

## 5 Conclusions

In summary, by studying present-day exposure–response associations, we demonstrate that there is no one-size-fits-all exposure metric for heat-related mortality research. Our results suggest that dry heat metrics (T_mean_ and Swmp20) dominate as the best-fit metric in Southern and Western Asia, Eastern Asia and Australia. AT, on the other hand, is the dominant heat stress metric for modelling warm-season heat-related mortality predominantly in Northern and Eastern Europe. However, using T_mean_ as the only deadly heat exposure metric regardless of the location, as most epidemiological studies do, results in similar country-level warm-season heat-related mortality in present-day climate, except for Spain.

## Supplementary Material

Additional supporting information can be found online in the Supporting Information section at the end of this article.

Supplementary information

## Data Availability

ERA5 data are available for download from the Copernicus Climate Change Service (C3S) Climate Data Store. Data used to generate the figures in this paper are publicly available from https://github.com/BrisClim/Heat-stress-mortality-paper. For further information about the MCC data, please contact Antonio Gasparrini (antonio.gasparrini@lshtm.ac.uk).
