# Supplementary material for: Optimal heat stress metric for modelling heat‐related mortality varies from country to country
Source: Int J Climatol. 2023 Jul 12;43(12):5553–68. Online ahead of print. doi: 10.1002/joc.8160 (PMC10410159; doi:10.1002/joc.8160)
Supplement: Supplementary file 1 [file JOC-43-5553-s001.docx]

Supplementary Information

**Optimal heat stress metric for modelling heat-related mortality varies from country to country**

Y. T. Eunice Lo^*^, Dann M. Mitchell, Jonathan R. Buzan, Jakob Zscheischler, ﻿Rochelle Schneider, Malcolm N. Mistry, Jan Kyselý, Éric Lavigne, Susana Pereira da Silva, Dominic Royé, Aleš Urban, Ben Armstrong, Multi-Country Multi-City (MCC) Collaborative Research Network, Antonio Gasparrini, Ana M. Vicedo-Cabrera^*^

*Corresponding authors. Email: eunice.lo@bristol.ac.uk, anamaria.vicedo@ispm.unibe.ch 

**This file includes:**

Figs. S1 to S2

Tables S1 to S2

**Figure S1.** Difference in attributable fraction between the best-fit metric and T_mean_ for selected countries. Only countries where the best-fit metric gives a noticeably better fit to warm-season mortality than T_mean_ are included. Countries are grouped by United Nations regions. Error bars show the 95% confidence intervals.

**Figure S2.** Fraction of deaths attributable to heat estimated from all studied heat stress metrics for all included MCC-defined countries. Error bars show the 95% confidence interval.

**Table S1.** Details of the included MCC-defined countries, including the number of locations in each country, and the time period used in this study.

| **MCC country** | **No. of locations** | **Period** |
| --- | --- | --- |
| Argentina | 3 | 2005-2015 |
| Australia | 3 | 1988-2009 |
| Brazil | 18 | 1997-2011 |
| Canada | 26 | 1986-2015 |
| Chile | 4 | 2004-2014 |
| China | 14 | 1996-2015 |
| Colombia | 5 | 1998-2013 |
| Costa Rica | 1 | 2000-2017 |
| Czech Republic | 4 | 1994-2015 |
| Ecuador | 2 | 2014-2018 |
| Estonia | 5 | 1997-2018 |
| Finland | 1 | 1994-2014 |
| France | 18 | 2000-2014 |
| Germany | 12 | 1993-2015 |
| Greece | 1 | 2001-2010 |
| Guatemala | 1 | 2009-2016 |
| Iran | 2 | 2002-2015 |
| Italy | 11 | 1987-2010 |
| Japan | 47 | 1979-2015* |
| Kuwait | 1 | 2000-2016 |
| Mexico | 10 | 1998-2014 |
| Moldova | 4 | 2001-2010 |
| Netherlands | 5 | 1995-2016 |
| Norway | 1 | 1979-2018^#^ |
| Panama | 1 | 2013-2016 |
| Paraguay | 1 | 2004-2019 |
| Philippines | 4 | 2006-2010 |
| Portugal | 5 | 1980-2018 |
| Peurto Rico | 1 | 2009-2016 |
| Romania | 8 | 1994-2016 |
| South Korea | 36 | 1997-2018 |
| Spain | 52 | 1990-2014 |
| Sweden | 3 | 1990-2016 |
| Switzerland | 8 | 1995-2013 |
| Taiwan | 3 | 1994-2014 |
| United Kingdom | 70 | 1990-2016 |
| USA | 210 | 1979-2006^&^ |
| Uruguay | 1 | 2012-2016 |
| Vietnam | 2 | 2009-2013 |

*MCC data span 1972-2015; ^#^ MCC data span 1969-2018; ^& ^MCC data span 1973-2006.

**Table S2.** Best-fit metric, attributable fraction estimated with the best-fit metric, attributable fraction attributed with T_mean_, and their 95% confidence intervals (in brackets) for each studied MCC-defined country.

| **MCC country** | **Best-fit metric** | **Attributable fraction, Best (%) (%)** | **Attributable fraction, T**_**mean**_ **(%)** |
| --- | --- | --- | --- |
| Argentina | T_mean_ | 2.98 (1.94–3.99) | 2.98 (1.94–3.99) |
| Australia | T_mean_ | 1.44 (0.51–2.29) | 1.44 (0.51–2.29) |
| Brazil | Swmp20 | 1.85 (1.42–2.27) | 1.95 (1.48–2.43) |
| Canada | AT | 1.12 (0.88–1.37) | 1.09 (0.86–1.34) |
| Chile | T_mean_ | 2.00 (0.77–3.24) | 2.00 (0.77–3.24) |
| China | Swmp20 | 1.92 (1.41–2.41) | 1.94 (1.48–2.41) |
| Colombia | AT | 0.42 (0.06–0.79) | 1.11 (0.20–1.95) |
| Costa Rica | T_w_ | 0.11 (-0.51–0.65) | 1.63 (-0.30–3.60) |
| Czech Republic | Swmp40 | 2.96 (2.43–3.50) | 3.13 (2.51–3.72) |
| Ecuador | T_w_ | 0.12 (-0.31–0.51) | 1.39 (-0.20–3.09) |
| Estonia | AT | 1.54 (0.82–2.25) | 1.58 (0.93–2.25) |
| Finland | AT | 1.78 (0.72–2.79) | 2.06 (1.08–3.03) |
| France | AT | 3.05 (2.77–3.33) | 3.18 (2.89–3.46) |
| Germany | Swmp20 | 3.13 (2.71–3.57) | 3.39 (3.00–3.80) |
| Greece | T_mean_ | 7.95 (6.37–9.43) | 7.95 (6.37–9.43) |
| Guatemala | T_w_ | 0.00 (-0.27–0.21) | 0.91 (-0.68–2.42) |
| Iran | T_mean_ | 2.42 (0.15–4.48) | 2.42 (0.15–4.48) |
| Italy | Swmp20 | 8.08 (7.28–8.86) | 8.32 (7.45–9.18) |
| Japan | Swmp20 | 1.45 (1.31–1.59) | 1.48 (1.34–1.63) |
| Kuwait | Swmp20 | 3.97 (1.23–6.51) | 4.86 (1.95–7.39) |
| Mexico | AT | 0.92 (0.61–1.22) | 1.31 (0.89–1.73) |
| Moldova | AT | 3.20 (1.50–4.85) | 3.07 (1.73–4.36) |
| Netherlands | T_mean_ | 2.12 (1.42–2.78) | 2.12 (1.42–2.78) |
| Norway | T_mean_ | 0.99 (-0.05–1.96) | 0.99 (-0.05–1.96) |
| Panama | AT | 1.18 (-1.60–3.64) | 1.59 (-1.01–4.15) |
| Paraguay | T_mean_ | 5.27 (3.42–7.10) | 5.27 (3.42–7.10) |
| Philippines | AT | 1.51 (0.70–2.28) | 2.33 (1.25–3.34) |
| Portugal | Swmp40 | 5.55 (4.91–6.21) | 5.90 (5.20–6.51) |
| Peurto Rico | T_w_ | 0.02 (-0.43–0.38) | 1.27 (-1.20–3.71) |
| Romania | AT | 5.14 (4.39–5.83) | 5.14 (4.33–5.95) |
| South Korea | AT | 1.08 (0.80–1.35) | 1.11 (0.85–1.36) |
| Spain | AT | 6.00 (5.61–6.38) | 7.05 (6.68–7.42) |
| Sweden | AT | 1.26 (0.71–1.76) | 1.37 (0.90–1.86) |
| Switzerland | Swmp60 | 3.22 (2.34–4.13) | 3.69 (2.92–4.49) |
| Taiwan | T_mean_ | 2.01 (1.12–2.88) | 2.01 (1.12–2.88) |
| United Kingdom | AT | 0.96 (0.71–1.20) | 0.94 (0.77–1.13) |
| USA | Swmp20 | 0.88 (0.80–0.96) | 0.90 (0.82–0.97) |
| Uruguay | AT | 3.77 (1.91–5.55) | 3.75 (1.95–5.46) |
| Vietnam | Swmp40 | 2.75 (1.12–4.49) | 3.38 (1.61–5.23) |

**Full list of MCC Collaborative Research Network authors**
Rosana Abrutzky^20^, Yuming Guo^21,22^, Shilu Tong^23,24,25^, Micheline de Sousa Zanotti Stagliorio Coelho^26^, Paulo Hilario Nascimento Saldiva^27^, Patricia Matus Correa^28^, Nicolás Valdés Ortega^28^, Haidong Kan^29^, Samuel Osorio^30^, Hans Orru^31^, Ene Indermitte^31^, Jouni J. K. Jaakkola^32,33^, Niilo Ryti^32,33^, Mathilde Pascal^34^, Alexandra Schneider^35^, Veronika Huber^36,37^, Klea Katsouyanni^38,39^, Antonis Analitis^38^, Alireza Entezari^22,40^, Fatemeh Mayvaneh^40^, Paola Michelozzi^41^, Francesca de'Donato^41^, Masahiro Hashizume^42^, Yoonhee Kim^43^, Barrak Alahmad^44^, Magali Hurtado Diaz^45^, César De la Cruz Valencia^45^, Ala Overcenco^46^, Danny Houthuijs^47^, Caroline Ameling^47^, Shilpa Rao^48^, Alfonso Diz Lois Palomares^48^, Xerxes Seposo^49^, Baltazar Nunes^50,51^, Joana Madureira^15,52,53^, Iulian-Horia Holobaca^54^, Ho Kim^55^, Whanhee Lee^56^, Aurelio Tobias^49,57^, Carmen Íñiguez^16,58^, Bertil Forsberg^59^, Christofer Åström^59^, Martina S. Ragettli^60,61^, Yue Leon Guo^62,63,64^, Shih-Chun Pan^63^, Valentina Colistro^65^, Antonella Zanobetti^44^, Joel Schwartz^44^, Tran Ngoc Dang^66,67^, Do Van Dung^67^, Yasushi Honda^68^, Francesco Sera^69^.

20 Universidad de Buenos Aires, Facultad de Ciencias Sociales, Instituto de Investigaciones Gino Germani
21 Department of Epidemiology and Preventive Medicine, School of Public Health and Preventive Medicine, Monash University, Melbourne, Australia
22 Climate, Air Quality Research Unit, School of Public Health and Preventive Medicine, Monash University, Melbourne, Australia
23 School of Public Health and Social Work, Queensland University of Technology, Brisbane, Australia
24 School of Public Health and Institute of Environment and Human Health, Anhui Medical University, Hefei, China
25 Shanghai Children’s Medical Centre, Shanghai Jiao-Tong University, Shanghai, China
26 Department of Pathology, Faculty of Medicine, University of São Paulo, São Paulo, Brazil 27 INSPER, São Paulo, Brazil
28 Department of Public Health, Universidad de los Andes, Santiago, Chile
29 Department of Environmental Health, School of Public Health, Fudan University, Shanghai, China
30 Department of Environmental Health, University of São Paulo, São Paulo, Brazil
31 Department of Family Medicine and Public Health, University of Tartu, Tartu, Estonia
32 Center for Environmental and Respiratory Health Research (CERH), University of Oulu, Oulu, Finland
33 Medical Research Center Oulu (MRC Oulu), Oulu University Hospital and University of Oulu, Oulu, Finland
34 Santé Publique France, Department of Environmental Health, French National Public Health Agency, Saint Maurice, France
35 Institute of Epidemiology, Helmholtz Zentrum München – German Research Center for Environmental Health (GmbH), Neuherberg, Germany
36 IBE-Chair of Epidemiology, LMU Munich, Munich, Germany
37 Department of Physical, Chemical and Natural Systems, Universidad Pablo de Olavide, Sevilla, Spain
38 Department of Hygiene, Epidemiology and Medical Statistics, National and Kapodistrian University of Athens, Greece
39 School of Population Health and Environmental Sciences, King's College, London, UK
40 Faculty of Geography and Environmental Sciences, Hakim Sabzevari University, Sabzevar 9617916487 Khorasan Razavi, Iran
41 Department of Epidemiology, Lazio Regional Health Service, Rome, Italy
42 Department of Global Health Policy, Graduate School of Medicine, University of Tokyo, Tokyo, Japan
43 Department of Global Environmental Health, Graduate School of Medicine, University of Tokyo, Tokyo, Japan
44 Department of Environmental Health, Harvard T.H. Chan School of Public Health, Harvard University, Boston, MA, USA
45 Department of Environmental Health, National Institute of Public Health, Cuernavaca, Morelos, Mexico
46 National Agency for Public Health of the Ministry of Health, Labour and Social Protection of the Republic of Moldova
47 National Institute for Public Health and the Environment (RIVM), Centre for Sustainability and Environmental Health, Bilthoven, Netherlands
48 Norwegian institute of Public Health, Oslo, Norway
49 School of Tropical Medicine and Global Health, Nagasaki University, Nagasaki, Japan
50 Department of Epidemiology, Instituto Nacional de Saúde Dr Ricardo Jorge
51 Centro de Investigação em Saúde Pública, Escola Nacional de Saúde Pública, Universidade NOVA de Lisboa, Lisboa, Portugal
52 EPIUnit - Instituto de Saude Publica, Universidade do Porto, Porto, Portugal
53 Laboratório para a Investigação Integrativa e Translacional em Saúde Populacional (ITR), Porto, Portugal
54 Faculty of Geography, Babes-Bolay University, Cluj-Napoca, Romania
55 Graduate School of Public Health, Seoul National University, Seoul, Republic of Korea
56 School of the Environment, Yale University, New Haven CT, USA
57 Institute of Environmental Assessment and Water Research (IDAEA), Spanish Council for Scientific Research (CSIC), Barcelona, Spain
58 Department of Statistics and Computational Research. Universitat de València, València, Spain
59 Department of Public Health and Clinical Medicine, Umeå University, Sweden
60 Swiss Tropical and Public Health Institute, Basel, Switzerland
61 University of Basel, Basel
62 Environmental and Occupational Medicine, National Taiwan University (NTU) College of Medicine and NTU Hospital, Taipei, Taiwan
63 National Institute of Environmental Health Science, National Health Research Institutes, Zhunan, Taiwan
64 Graduate Institute of Environmental and Occupational Health Sciences, NTU College of Public Health, Taipei, Taiwan
65 Department of Quantitative Methods, School of Medicine, University of the Republic, Montevideo, Uruguay
66 Institute of Research and Development, Duy Tan University, Da Nang, Vietnam
67 Department of Environmental Health, Faculty of Public Health, Department of Environmental Health, University of Medicine and Pharmacy at Ho Chi Minh City, Ho Chi Minh City, Vietnam 68 Center for Climate Change Adaptation, National Institute for Environmental Studies, Tsukuba, Japan
69 Department of Statistics, Computer Science and Applications "G. Parenti", University of Florence, Florence, Italy.
